# CD151 mediates netrin‐1‐induced angiogenesis through the Src‐FAK‐Paxillin pathway

**DOI:** 10.1111/jcmm.12939

**Published:** 2016-08-25

**Authors:** Xiaosheng Yang, Shiting Li, Jun Zhong, Wenchuan Zhang, Xuming Hua, Bin Li, Hui Sun

**Affiliations:** ^1^ Department of NeurosurgeryXinHua HospitalShanghai JiaoTong University School of MedicineShanghaiChina

**Keywords:** netrin‐1, CD151, FAK/Src signalling, angiogenesis

## Abstract

Crosstalk between the nervous and vascular systems is important during development and in response to injury, and the laminin‐like axonal guidance protein netrin‐1 has been studied for its involvement in angiogenesis and vascular remodelling. In this study, we examined the role of netrin‐1 in angiogenesis and explored the underlying mechanisms. The effect of netrin‐1 on brain tissues and endothelial cells was examined by immunohistochemistry and western blotting in a middle cerebral artery occlusion model and in human umbilical vein endothelial cells. Cell proliferation and cell cycle progression were assessed by the MTT assay and flow cytometry, and the Transwell and tube formation assays were used to examine endothelial cell motility and function. Netrin‐1 up‐regulated CD151 and VEGF concomitant with the activation of focal adhesion kinase (FAK), Src and Paxillin *in vitro* and *in vivo* and the induction of cell proliferation, migration and tube formation *in vitro*. Silencing of CD151 abolished the effects of netrin‐1 on promoting cell migration and tube formation mediated by the activation of FAK/Src signalling. Netrin‐1 promoted angiogenesis *in vitro* and *in vivo* by activating the FAK/Src/Paxillin signalling pathway through a mechanism dependent on the expression of the CD151 tetraspanin, suggesting the existence of a netrin‐1/FAK/Src/CD151 signalling axis involved in the modulation of angiogenesis.

## Introduction

Stroke is one of the most common causes of death and adult disability, severely affecting the quality of life of survivors 1. The loss of function associated with stroke is in part compensated by the re‐establishment of connectivity among surviving neurons, and this process is primarily attributed to the induction of axonal sprouting and outgrowth and the regeneration of brain vasculature 2. Netrin‐1 is a 60–80 kDa laminin‐like protein originally identified as an axon guidance molecule during neural development in *Caenorhabditis elegans* 3. Netrin‐1 belongs to a family of three secreted netrins whose activity is mediated by several receptors. Netrin‐1 functions during brain development by establishing a gradient that attracts growing axons expressing netrin receptors to the midline of the central nervous system 4. Netrin‐1 also functions as a vascular mitogen by promoting endothelial and vascular smooth muscle cell proliferation, migration and adhesion, and it accelerates neovascularization in several *in vitro* and *in vivo* models 5; however, it has also been shown to have anti‐angiogenic effects 6. Netrin‐1 inhibits inflammation and apoptosis, promoting repair after ischemic brain injury by increasing angiogenesis 5, 7. Netrin‐1 is up‐regulated in tumours and acts as an oncogene, promoting tumour progression and angiogenesis in several cancers, including breast cancer, melanoma, colorectal cancer, non‐small cell lung cancer, pancreatic adenocarcinoma, and glioblastoma among others, and its oncogenic effect is mediated by several signalling pathways 8, 9, 10, 11, 12.

Crosstalk between the nervous and vascular systems plays a role during development and in the response to injury 13. Vascular connections are formed to prevent ischemic damage to neural tissues, and conversely neuroregulatory signals play a role in vascular development. VEGF drives angiogenesis in many tissues, and it also plays a role in neural development, axonal outgrowth and neural progenitor attraction, and different neural regulatory factors are involved in both nervous system development and vascular morphogenesis and differentiation 14, 15, 16.

The development and integrity of tissues are partly dependent on the interaction between cells and the extracellular matrix (ECM), and defects in ECM proteins or deregulated cell adhesion underlie the pathogenesis of many diseases 17. Cell adhesion and spreading is mediated by the recruitment of Src kinase to adhesion sites through the interaction of its SH2 domain with the autophosphorylation site of focal adhesion kinase (FAK), leading to the phosphorylation of focal adhesion proteins such as FAK itself, paxillin, and p130Cas 18, 19. These phosphorylated residues act as docking sites for proteins that regulate the activity of Rho family GTPases to advance cell protrusion and spreading. FAK is a non‐receptor protein kinase that regulates several biological processes including cell motility, differentiation, angiogenesis and survival in addition to its role in modulating cell adhesion and migration *via* the FAK/Src/Paxillin cascade 20. Dynamic interactions between the ECM and the actin cytoskeleton during cell motility are mediated by integrins, a group of cell surface αβ heterodimers that associate with different matrix ligands such as fibronectin, collagen and laminin 21. Integrins form complexes with tetraspanins, which are ubiquitously expressed membrane proteins involved in many cellular processes including cell proliferation, migration and invasion. The CD151 tetraspanin is expressed in epithelial, endothelial and fibroblastic cells, and it interacts with the α5β1 integrin to regulate cell adhesion and integrin‐dependent cell migration 22. The α6β4 and α3β1 integrins regulate the migration of epithelial cells through a direct interaction of these integrins with the C‐terminal domain of netrin‐1, and the modulatory activity of CD151 is dependent on its association with the α3β1 and α6β4 integrins 23, 24. CD151 was shown to promote neovascularization and angiogenesis in a rat model of hindlimb ischemia and to improve ventricular function after myocardial infarction in rats 25, 26.

In the present study, we used a rat middle cerebral artery occlusion (MCAO) model and human umbilical vein endothelial cells (HUVECs) as a model of vascular endothelial cells to examine the effect of netrin‐1 on angiogenesis and endothelial cell proliferation, migration, and tube formation and explored the underlying mechanisms.

## Experimental protocols

### Animal model and experimental protocols

All animal procedures were performed according to a protocol approved by the Institutional Animal Care and Use Committee of Shanghai Jiao Tong University, China. For *in vivo* experiments, a total of 36 rats were used with six rats per group.

Small interfering RNA (siRNA) against CD151 (Si‐CD151) was purchased from Dharmacon Thermo Scientific and administered by intracerebroventricular (ICV) injection at a concentration of 500 ng/l nmol in 5 μl according to previously published protocols 27, 28. Each injection occurred at a rate of 0.5 μl/min. using a 30 G stainless steel needle on a 10 μl Hamilton syringe, held by the micromanipulator on the stereotactic apparatus. The needle remained in place for another 10 min. after the injection to allow for sufficient diffusion.

To test whether the delivery of netrin‐1 protein affects acute brain ischemic injury in rats, male Sprague–Dawley rats were treated by ICV injection with one dose of recombinant chicken netrin‐1 (200 ng/ml, R&D Systems, Minneapolis, MN, USA) at 1 hr after reperfusion.

### MCAO

Stroke was induced unilaterally in rats by the distal middle cerebral artery occlusion (dMCAO) method under isoflurane (1.5%)/O_2_ (30%)/N_2_O (68.5%) anaesthesia as previously described 29. In brief, the main trunk of the left MCA was ligated just underneath the rhinal fissure with a 10‐0 suture, and the bilateral common carotid arteries (CCA) were occluded for 60 min. with 4‐0 sutures. The sutures were then removed to restore blood flow, and the cervical incision was closed.

Operated rats were then divided into groups based on whether they received treatment with siRNA‐CD151 or siRNA‐control into the brain 48 hrs before MCAO, and treated with recombinant chicken netrin‐1 (200 ng/ml, R&D Systems) in a total volume of 1 ml of PBS containing 1% BSA.

### Real‐time reverse transcription‐polymerase chain reaction (RT‐PCR) analysis

Total RNA was extracted from brain tissues using the TRIzol reagent (Invitrogen, Carlsbad, CA, USA) and treated with RNase‐free DNase I. Total RNA (100 ng) was used as template for real‐time PCR analysis. Reactions contained 1 μl RNA, 9 μl of LightCycler FastStart DNA Master SYBR Green I mix (Roche, Indianapolis, IN, USA) and gene‐specific forward and reverse PCR primers. The sequences of the primers were as follows: rat CD151 upstream, 5′‐GTACTTCATCCTGCTCCTCA‐3′; and downstream, 5′‐CAGCCACCTTCCACTTTAT‐3′; rat VEGF upstream, 5′‐ATCCAATCGAGACCCTGGTG‐3′ and downstream, 5′‐ATCTCTCCTATGTGCTGGCC‐3′; human CD151, upstream,5′‐GAGGTCTATGGGTGAGTTCAACGAG‐3′ and downstream, 5′‐AATTCCTAGGCGTAGTC‐3′, human VEGF upstream, 5′‐ATCCAATCGAGACCCTGGTG‐3′ and downstream, 5′‐ATCTCTCCTATGTGCTGGCC‐3′. PCR conditions were as follows: 5 min. at 95°C, followed by 40 cycles of 30 sec. at 95°C, 30 sec. at 57°C, and 30 sec. at 72°C. Ct values were normalized to the GAPDH gene as a cDNA loading control and changes were calculated relative to controls.

### Immunohistochemistry analysis

Brain sections were incubated with 10% serum for 1 hr to block non‐specific binding, washed with PBS, and then incubated with anti‐CD31 primary antibody (1:150 dilution, Santa Cruz Biotechnology Inc., Santa Cruz, CA, USA) in PBS at 4°C overnight. Biotin‐conjugated secondary antibody, avidin‐biotin enzyme reagent (Vector Laboratories, Inc., Burlingame, CA, USA), and diaminobenzidine (DAB) were used to visualize the signal. After counterstaining with hematoxylin, the sections were dehydrated for further microscopic study.

Control rats were treated by ICV injection with 1 ml of PBS containing 1% BSA at 1 hr after reperfusion. Netrin‐1‐treated rats received ICV injection of one dose of recombinant chicken netrin‐1 (200 ng/ml, R&D Systems) at 1 hr after reperfusion.

### Western blotting

Brain samples were collected at 24 hrs after MCAO and homogenized in lysis buffer. The protein concentrations were determined using the bicinchoninic acid (BCA) protein assay kit (Pierce, Rockford, IL, USA). Equal amounts of total protein (30 μg) were loaded onto 5–10% gradient gels for electrophoresis, followed by transfer to polyvinylidene difluoride membranes. Membranes were blocked in 2% BSA followed by incubation with the following primary antibodies (all dilutions 1:100; Santa Cruz Biotechnology): anti‐Src, anti‐phospho‐Src, anti‐FAK, anti‐phospho‐FAK, anti‐netrin‐1.

### Cell viability

HUVECs were serum‐starved for 24 hrs and cell proliferation was assessed using a CCK‐8 kit (Dojindo, Kumamoto, Japan). Briefly, HUVECs were suspended and seeded into a 96‐well plate. Netrin‐1 was directly added to the culture medium. After 24–96 hrs or 48 hrs of culture, cells were incubated with 100 μl of 10% CCK‐8 reagent for 1 hr at 37°C. The colour reaction was measured at 450 nm with a BioRad ELISA reader (Richmond, CA, USA).

### Flow cytometry

HUVECs were grown under the above‐described conditions in 35‐mm dishes until confluent. Netrin‐1 was added to the medium at concentrations of 0, 12.5, 50, or 100 ng/ml. Incubation was continued for 48 hrs at 37°C. Cells were removed using 0.05% trypsin in PBS, pelleted (centrifugation at 300 g for 5 min.), washed twice in 1% BSA in PBS, resuspended in 1% BSA in PBS, and fixed in 70% cold ethanol. A flow cytometer (FACScan; BD Biosciences, Franklin Lakes, NJ, USA) was used to acquire all data.

### Transwell and tube formation assay

For the cell migration assay, HUVECs were suspended in fresh serum‐free medium and seeded in the upper chambers of Transwell plates to a density of 5 × 10^3^ cells per chamber. The lower chambers contained fresh medium with 10% foetal calf serum (FCS). After 12 hrs of incubation at 37°C, cells remaining on the upper surface of the membrane were removed using a swab, whereas the cells migrated to the lower membrane surface were fixed with 4% PFA and stained with 0.1% crystal violet solution. Cells were counted in 25 random fields under a microscope. The assay was carried out in duplicate and repeated three times for quantitative analysis.

In the tube formation assay, 96‐well culture plates were coated with Matrigel (BD Biosciences) to a total volume of 60 μl per well and allowed to solidify for 30 min. at 37°C. HUVECs were serum‐starved for 24 hrs and seeded to a density of 1 × 10^4^ cells per well. Netrin‐1 was added directly to the HUVEC suspensions in complete 1640 medium. Cells were incubated at 37°C for 6 hrs. Tube formation was observed under an inverted microscope (Eclipse Model TS100; Nikon, Japan). At least five fields in each well were examined. The relative length of tubes was quantified using Image J software (USA).

### Statistical analysis

Data are presented as the mean ± S.E.M. Data were analysed using Student's *t*‐test and *P* < 0.05 were considered statistically significant. Statistical analyses were performed with one‐way anova.

## Results

### Netrin‐1 induces angiogenesis and activates FAK/Src signalling *via* CD151 in the rat brain

**Figure 1:**
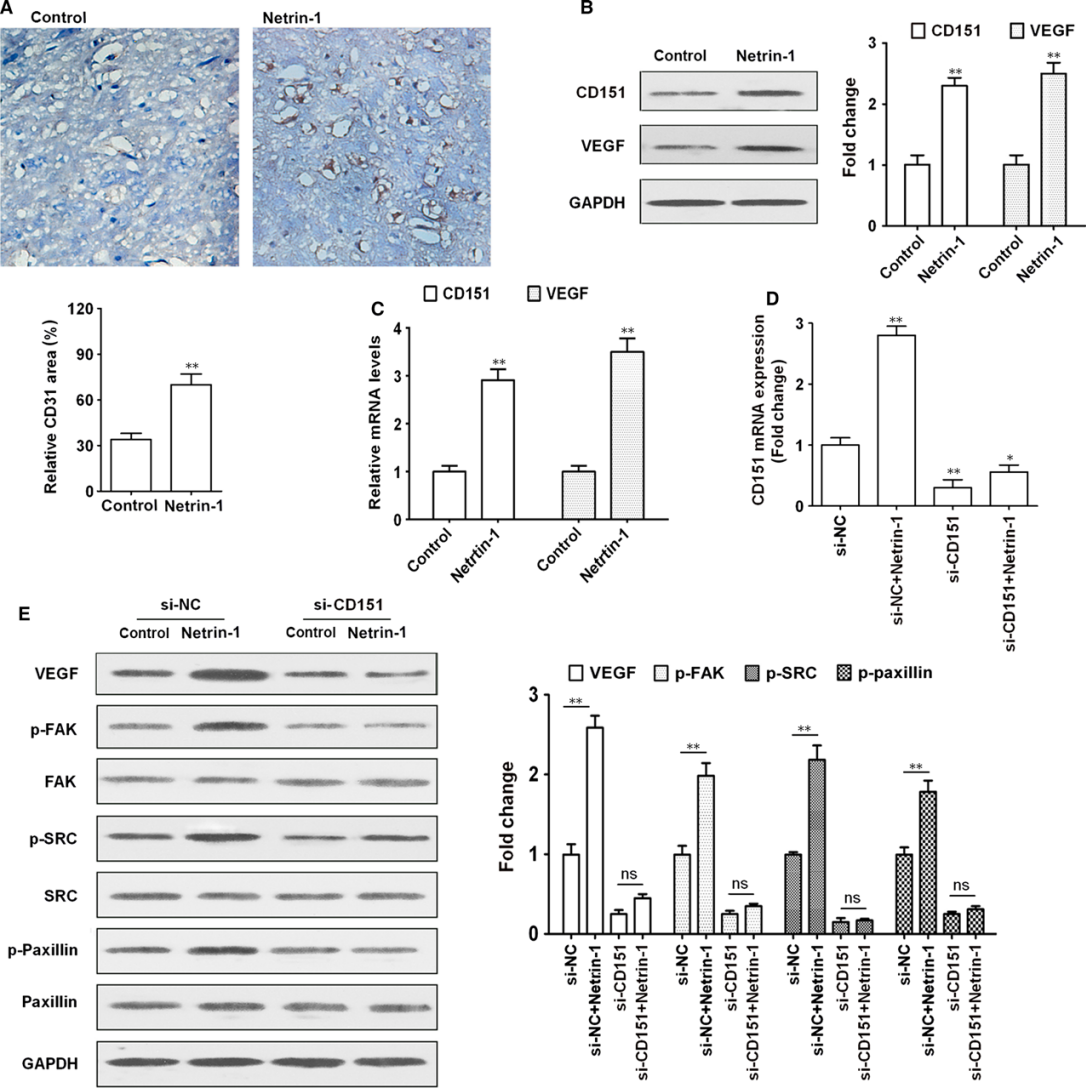
Netrin‐1 induces angiogenesis *via* CD151. (**A**) Photomicrographs showing CD‐31 positive microvessels in the rat brain. The control or netrin‐1 (200 ng/ml) was injected subcutaneously into rats in the corresponding groups. Images are shown at ×200 magnification. (**B**) Western blot analysis of CD151 and VEGF expression in brain tissues and densitometric quantification normalized to controls. (**C**) The relative expression of CD151 and VEGF was examined by qRT‐PCR. (**D** and **E**) Rats were transfected with siRNA against CD151 or siRNA‐control 48 hrs before MCAO. After 24 hrs MCAO, the relative expression of CD151 was examined by qRT‐PCR and the results were normalized to the si‐NC. (**E**)The expression of VEGF, FAK/pFAK, SRC, pSRC and paxillin/p‐paxillin in the rat brain was examined by western blotting using GAPDH expression as a loading control. *n* = 6 per group *P < 0.05; **P < 0.01. FAK, focal adhesion kinase; VEGF, vascular endothelial growth factor.

The effect of netrin‐1 on angiogenesis was assessed by immunohistochemical staining of rat brain sections with anti‐CD31 antibody. CD31 is an endothelial cell adhesion molecule that is used to determine the presence of endothelial cells in tissue sections, which reflects the degree of angiogenesis. Rats treated with netrin‐1 showed an approximately 2.5‐fold increase in the CD31 positive area, indicating that netrin‐1 promoted angiogenesis (Fig. 1A). Western blot and real‐time RT‐PCR analyses of brain tissues showed that netrin‐1 significantly up‐regulated VEGF and CD151 in the rat brain at the protein and mRNA levels (Fig. 1B and C). To further examine the role of CD151 in the effect of netrin‐1, rats were injected with siRNA (against CD151 or siRNA‐control at 48 hrs before MCAO. Operated rats were then divided into groups based on ICV injection of recombinant chicken netrin‐1 (200 ng/ml) in a total volume of 1 ml PBS containing 1% BSA at 1 hr after reperfusion. qRT‐PCR analysis of brain tissues showed that netrin‐1 up‐regulated CD151 and this effect was abolished in rats treated with siRNA against CD151 (Fig. 1D). Netrin‐1 up‐regulated VEGF and activated the FAK/Src/Paxillin cascade, as indicated by the up‐regulation of the phosphorylated forms of FAK, Src and Paxillin in netrin‐1‐treated tissues (Fig. 1E). Silencing of CD151 abolished the effect of netrin‐1 on the up‐regulation of VEGF and the activation of the FAK/Src pathway, suggesting that CD151 mediates the effects of netrin‐1 on the brain in a MCAO model.

### Netrin‐1 promotes cell proliferation and cell cycle progression

**Figure 2:**
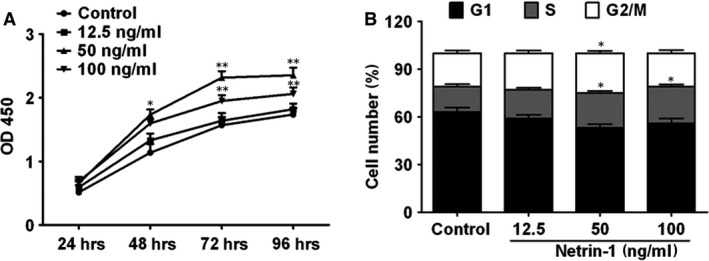
Netrin‐1 promotes cell proliferation and cell cycle progression. (**A**) HUVEC cell viability in response to different concentrations of netrin‐1 for 24–96 hrs was assessed by the CCK8 assay. (**B**) Cell cycle detected by flow cytometric analysis in cells exposed to different concentrations of netrin‐1 for 48 hrs. All experiments were done in triplicate. Mean ± S.E.M. was calculated from independent experiments. *P < 0.05; **P < 0.01.

The effect of netrin‐1 on cell proliferation and cell cycle progression was analysed in HUVECs treated with increasing concentrations of netrin‐1 for 24–96 hrs. The results of the CCK8 assay showed that netrin‐1 promoted cell proliferation in a dose‐dependent manner (Fig. 2A). Flow cytometry analysis showed that netrin‐1 significantly induced cell cycle progression at doses of 50 and 100 ng/ml (Fig. 2B).

### Netrin‐1 promotes endothelial cell migration and tube formation

**Figure 3:**
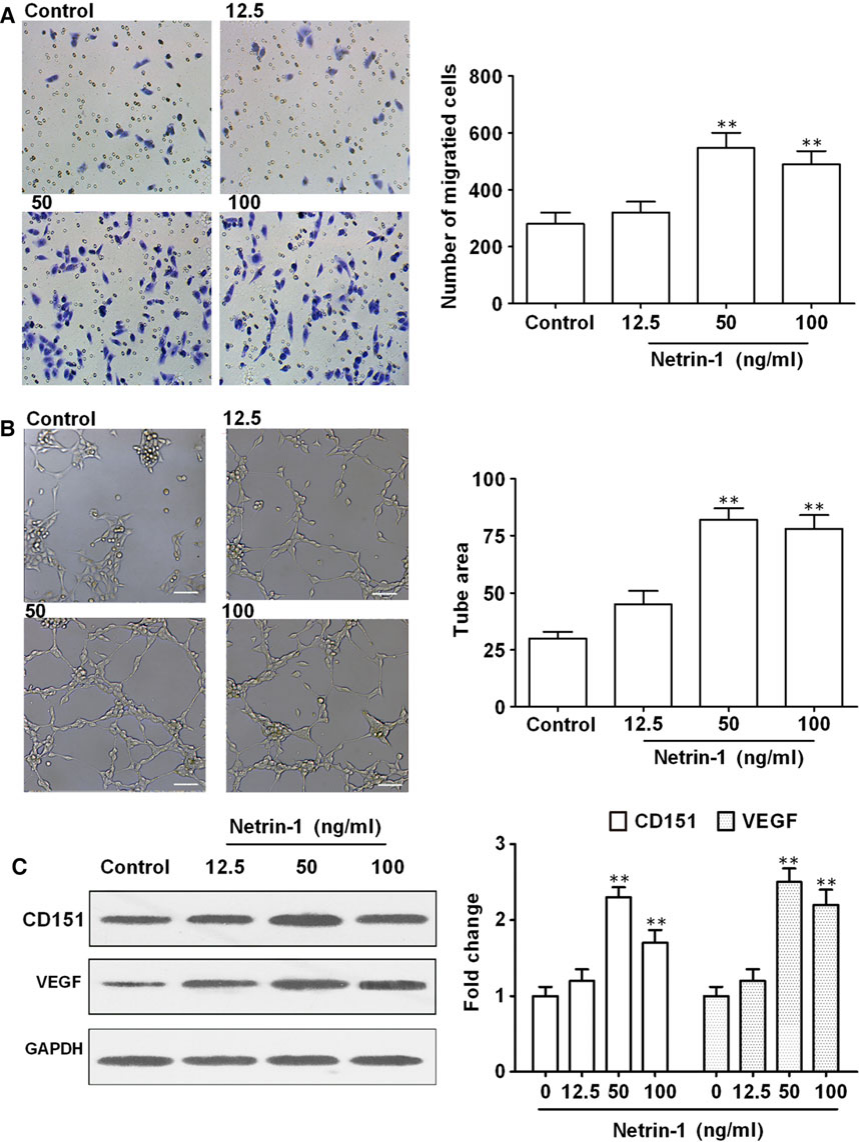
Effects of netrin‐1 on cell migration. (**A**) HUVEC cells were treated with increasing concentrations of netrin‐1 and cell migration was assessed using the Transwell assay, and (**B**) tube formation was assessed using a tube formation assay. (**C**) The expression of CD151 and VEGF was determined by western blotting and normalized to the untreated controls. Scale bar, 50 μm. All experiments were done in triplicate. Mean ± S.E.M. was calculated from independent experiments. **P < 0.01. VEGF, vascular endothelial growth factor.

To further examine the effects of netrin‐1 on angiogenesis, HUVECs were treated with different concentrations of netrin‐1 and cell migration and tube formation, which are critical steps in the formation of new blood vessels, were assessed. The results showed that netrin‐1 significantly induced cell migration and tube formation at concentrations of 50 and 100 ng/ml (Fig. 3A and B), indicating that low concentrations of netrin‐1 promote angiogenesis. The number of EC branch points and tube length were quantified in Figure S1. Furthermore, these effects were accompanied by the significant up‐regulation of CD151 and VEGF by approximately 2.5‐fold in response to 50 ng/ml of netrin‐1, as determined by western blotting and densitometric quantification (Fig. 3C), which suggested that the effect of netrin‐1 on promoting angiogenesis is mediated by CD151. Also, these results were confirmed by qRT‐PCR (Fig. S2).

### Netrin‐1 promotes angiogenesis and activates FAK/Src signalling in a CD151‐dependent manner

**Figure 4:**
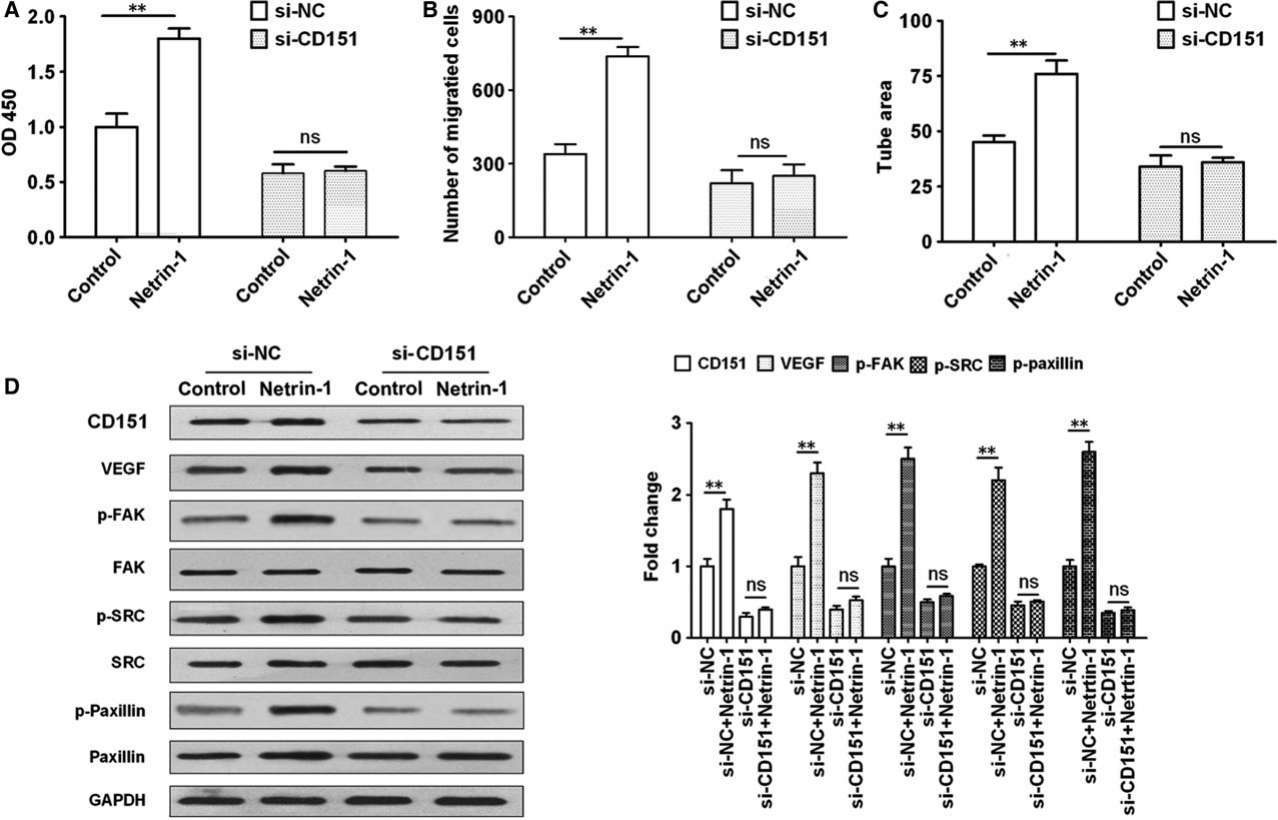
The effects of netrin‐1 were dependent on the expression of CD151. HUVECs were treated with si‐NC or si‐CD151 and cell proliferation, migration and tube formation in response to netrin‐1 treatment (50 ng/ml) were assessed by (**A**) CCK8 assay, (**B**) Transwell assay and (**C**) tube formation assay. (**D**) Western blot analysis of VEGF and FAK signalling proteins in HUVECs treated with si‐NC or si‐CD151. All experiments were performed in triplicate. The mean ± S.E.M. was calculated from independent experiments. **P < 0.01; ‘NS’ indicates no significant changes observed. FAK, focal adhesion kinase; VEGF, vascular endothelial growth factor.

To further examine the relation between netrin‐1 and CD151, the effects of netrin‐1 on HUVEC proliferation, migration, tube formation and FAK/Src signalling were assessed in the presence or absence of siRNA against CD151. Silencing of CD151 abolished the effects of netrin‐1 on increasing proliferation, migration and tube formation in HUVECs (Fig. 4A–C). Consistently, the effects of netrin‐1 on the up‐regulation of VEGF and the activation of FAK, Src and Paxillin were abolished by silencing of CD151 (Fig. 4D). Taken together, these results indicated that netrin‐1 promotes endothelial cell migration and angiogenesis by activating FAK/Src signalling *via* a mechanism dependent on the expression of CD151.

## Discussion

Netrin‐1 stimulates proliferation and migration and promotes the adhesion of endothelial cells and vascular smooth muscle cells, playing an important role in the induction of angiogenesis. The mechanism by which netrin‐1 induces angiogenesis was proposed to involve the activation of its receptor deleted in colorectal cancer (DCC), which triggers the activation of extracellular signal‐regulated kinase (ERK)1/2, leading to an increase in the production of endothelial nitric oxide and the induction of endothelial cell migration and growth 30. Because of its role in promoting angiogenesis, netrin‐1 has been researched in relation to ischemic coronary artery disease and cerebral ischemia and shown to reduce ischemia/reperfusion injury to improve heart function and promote long‐term functional recovery after focal cerebral ischemia 31, 32. However, at high concentrations, netrin‐1 has also been shown to exert antiangiogenic effects mediated by binding to UNC5b in endothelial cells, which inhibits sprouting angiogenesis 33. In the present study, we showed that at low concentrations, netrin‐1 promotes angiogenesis in a rat MCAO model and endothelial cell proliferation, migration and tube formation by up‐regulating VEGF and activating the FAK/Src/Paxillin pathway *via* a mechanism dependent on CD151.

We generated an MCAO model to examine the effects of netrin‐1 and explore the underlying mechanisms, and found that netrin‐1 up‐regulated VEGF and CD151, and activated FAK, Src and Paxillin in the rat brain, concomitant with an increase in cell proliferation and the induction of cell cycle progression and angiogenesis. The pro‐angiogenic effects of netrin‐1 have been demonstrated in different models and tissues. Netrin‐1 accelerates neovascularization in a model of hindlimb ischemia, and exogenous netrin‐1 induces neovascularization in the mouse brain and increases the density of functionally competent blood vessels in the infarcted heart 16, 34, 35. Netrin‐1 is expressed in non‐neural tissues during early murine development, when angiogenesis is active, suggesting its involvement in angiogenesis 36. Furthermore, netrin‐1 not only stimulates proliferation, migration and adhesion of vascular cells similar to the effect of VEGF, but it also promotes the angiogenic activity of VEGF *in vitro* and *in vivo*. Despite extensive evidence supporting the role of netrin‐1 in the regulation of vascular morphogenesis, its dual role in angiogenesis, similar to its effect on axonal guidance, and the receptors mediating its pro‐ and anti‐angiogenic activities remain unclear.

Using HUVECs as a model of vascular endothelial cells, we showed that low concentrations of netrin‐1 (50 or 100 ng/ml) promoted cell proliferation, migration and tube formation and up‐regulated CD151 and VEGF. Previous studies have shown that the pro‐ or anti‐angiogenic effects of netrin‐1 depend on its concentration and the receptor it interacts with 37. At high concentrations, netrin‐1 (1000 or 2000 ng/ml) inhibits angiogenesis by binding to the UNC5B receptor, whereas at low concentrations, netrin‐1 (50 or 200 ng/ml) promotes angiogenesis by binding to CD146, suggesting that the role of netrin‐1 may depend on the levels of specific receptors in relation to its own concentration 33, 37. Tu *et al*. identified the endothelial transmembrane protein CD146 (also known as melanoma cell adhesion molecule) as a receptor for netrin‐1 and showed that their interaction is necessary for netrin‐1‐induced endothelial cell activation and VEGF signal transduction 37. In mammals, netrin‐1 has six receptors belonging to two families. DCC and neogenin are members of the immunoglobulin superfamily, whereas the UNC5 family includes four transmembrane receptors 38. The best studied receptors for netrin‐1 are DCC and UNC5H2, although their roles in the brain and, in particular, in neovascularization and angiogenesis have not been explored in detail 39. We showed that the effect of netrin‐1 on promoting cell proliferation, migration and tube formation and its up‐regulation of VEGF and activation of FAK/Src signalling were abolished in CD151 knockdown cells, suggesting that the pro‐angiogenic effect of netrin‐1 was dependent on CD151. CD151 is expressed at high levels in endothelial cells and its role in the induction of cell migration and adhesion and in promoting angiogenesis has been demonstrated extensively 40. CD151 was shown to promote endothelial cell proliferation and tube formation by up‐regulating eNOS *via* the PI3K/Akt pathway 26. In HUVECs, CD151 promotes cell proliferation, migration and angiogenesis by activating the c‐Met signalling pathway and increasing the levels of nitric oxide, vascular cell adhesion molecule‐1 and VEGF 40. The results of the present study suggest an association between netrin‐1 and CD151; however, further experiments are necessary to determine whether netrin‐1 interacts directly with CD151 and the exact effects of their interaction.

In the present study, we showed that the effects of netrin‐1 on promoting angiogenesis were mediated by the activation of FAK/Src/Paxillin signalling *in vivo* and *in vitro*. FAK and Src are activated by ECM components *via* integrin receptors 41. In endothelial cells, crosstalk between integrins and VEGF receptor signalling plays a role in angiogenesis 42. Treatment of endothelial cells with VEGF induces the formation of a complex between FAK and αvβv integrin in a Src‐dependent manner 43. These studies support the role of the interaction between the ECM and growth factors in the regulation of integrin‐mediated endothelial cell adhesion and survival during angiogenesis. We showed that netrin‐1 up‐regulated CD151 in brain tissues and HUVECs in association with the up‐regulation of VEGF and the induction of cell migration and tube formation. CD151 promotes cell migration and invasion in a FAK‐dependent manner and induces neovascularization and angiogenesis through the activation of FAK‐dependent signalling pathways 44, 45. Our results showing that the effect of netrin‐1 on angiogenesis mediated by FAK/Src signalling was dependent on the expression of CD151 in the brain and in endothelial cells suggest a possible signalling axis involving netrin‐1 and the CD151 tetraspanin resulting in the activation of FAK/Src/Paxillin and the induction of angiogenesis. Although further studies are necessary to verify the interaction between these molecules and their effect on signalling pathways regulating angiogenesis and neovascularization, our results suggest potential novel therapeutic targets for the treatment of ischemic stroke and potentially other vascular disorders.

## Author contributions

Dr. Hui Sun designed research; Dr. Xiaosheng Yang and Dr. Hui Sun performed research; Dr. Shiting Li, Jun Zhong, Wenchuan Zhang, Xuming Hua and Bin Li contributed new reagents/analytic tools and analyzed data; and Dr Hui Sun wrote the paper.

## Conflict of interest

The authors confirm that there are no conflicts of interest.

## Supporting information


**Fig. S1** HUVEC cells were treated with increasing concentrations of netrin‐1. The number of EC branch points and tube length were quantified.

**Fig. S2** HUVEC cells were treated with increasing concentrations of netrin‐1 and the expression of VEGF and CD151 was determined by real‐time PCR.

**Fig. S3** HUVEC cells were treated with increasing concentrations of netrin‐1. The expression of CD151 was determined by real‐time RT‐PCR and normalized to the untreated controls.
