# Supplementary figures and images for: CD151 mediates netrin‐1‐induced angiogenesis through the Src‐FAK‐Paxillin pathway
Source: J Cell Mol Med. 2016 Aug 25;21(1):72–80. doi: 10.1111/jcmm.12939 (PMC5192806; doi:10.1111/jcmm.12939)

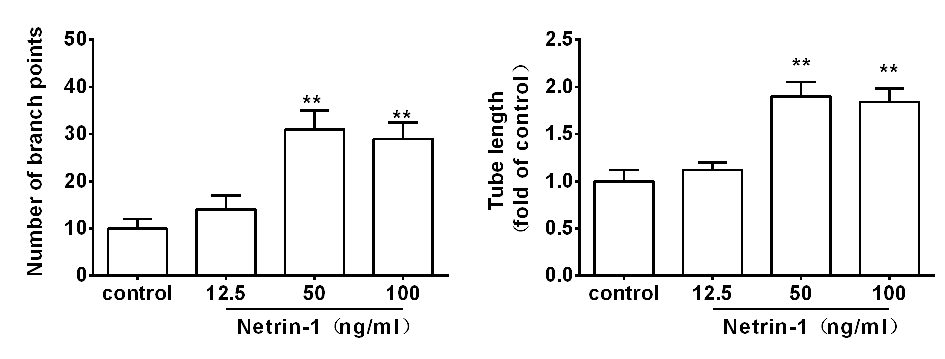

Supplement: Supplementary file 1 — Fig. S1 HUVEC cells were treated with increasing concentrations of netrin‐1. The number of EC branch points and tube length were quantified. [file JCMM-21-72-s001.tif]

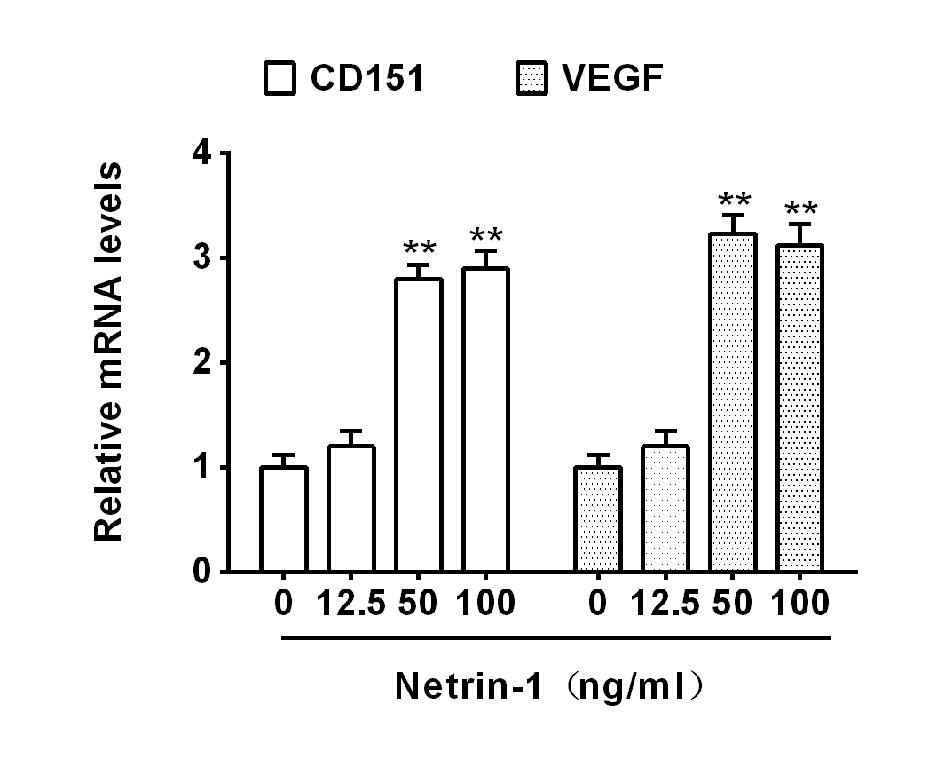

Supplement: Supplementary file 2 — Fig. S2 HUVEC cells were treated with increasing concentrations of netrin‐1 and the expression of VEGF and CD151 was determined by real‐time PCR. [file JCMM-21-72-s002.tif]

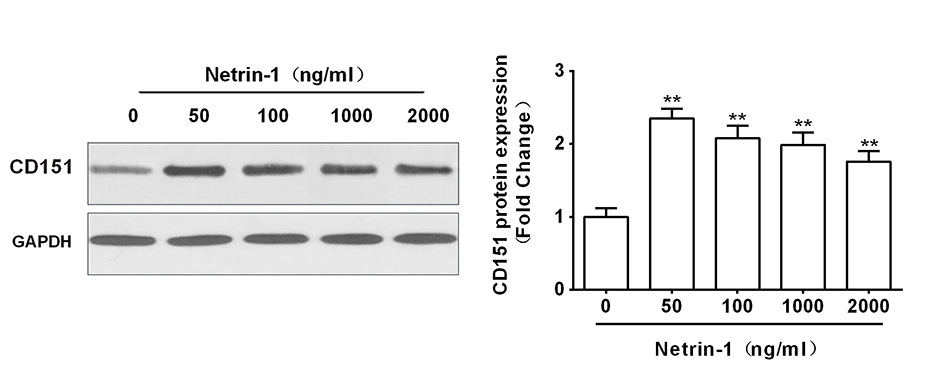

Supplement: Supplementary file 3 — Fig. S3 HUVEC cells were treated with increasing concentrations of netrin‐1. The expression of CD151 was determined by real‐time RT‐PCR and normalized to the untreated controls. [file JCMM-21-72-s003.tif]
